# Mr. Bransby Cooper—the Astley Cooper Prize—Castleton Medical College, Vt.

**Published:** 1842-04-30

**Authors:** 


MISCELLANEOUS NOTICES.

Mr. Bransby Cooper, nephew to Sir Astley, and Surgeon to Guy's Hospital, has been made a Baronet.

*The Astley Cooper Prize.*—The subject for the first triennial prize of three hundred pounds, under the will of the late Sir Astley Cooper, will be the Structure and Uses of the *Thymus Gland.* "The Essays or Treatises written for such prize shall contain original experiments and observations, which shall not have been previously published, and shall be illustrated by preparations or drawings, which shall be added to the Museum of Guy's Hospital, and, together with the copy-right of the work, become the property of the Hospital." The Essays written in English or Latin, distinguished by a motto, and accompanied by a sealed envelope, containing the name and address of the writer, are to be sent on or before the first of January, 1844, to the Physicians and Surgeons of Guy's Hospital, London. The competition for the prize is, we infer, open to American Physicians.

*Castleton Medical College, Vermont.*—We learn that this institution has nowa class of upwards of sixty.

